# Amidoamine Oxide Surfactants as Low-Molecular-Weight Hydrogelators: Effect of Methylene Chain Length on Aggregate Structure and Rheological Behavior

**DOI:** 10.3390/gels9030261

**Published:** 2023-03-22

**Authors:** Rie Kakehashi, Naoji Tokai, Makoto Nakagawa, Kazunori Kawasaki, Shin Horiuchi, Atsushi Yamamoto

**Affiliations:** 1 Surfactant Laboratory, Osaka Research Institute of Industrial Science and Technology, Osaka 536-8553, Japan; 2 Biomedical Research Institute, National Institute of Advanced Industrial Science and Technology (AIST), Ikeda 563-8577, Japan; 3 Nanomaterials Research Institute, National Institute of Advanced Industrial Science and Technology (AIST), Tsukuba 305-8565, Japan; 4 Faculty of Environmental Studies, Tottori University of Environmental Studies, Tottori 689-1111, Japan

**Keywords:** amidoamine oxide, amine oxide surfactant, low-molecular-weight gelator, hydrogel, gelation temperature, aggregate structure, methylene chain length, electron microscopy, rheology control, supramolecular gel

## Abstract

Rheology control is an important issue in many industrial products such as cosmetics and paints. Recently, low-molecular-weight compounds have attracted considerable attention as thickeners/gelators for various solvents; however, there is still a significant need for molecular design guidelines for industrial applications. Amidoamine oxides (AAOs), which are long-chain alkylamine oxides with three amide groups, are surfactants that act as hydrogelators. Here, we show the relationship between the length of methylene chains at four different locations of AAOs, the aggregate structure, the gelation temperature *T*_gel_, and the viscoelasticity of the formed hydrogels. As seen from the results of electron microscopic observations, the aggregate structure (ribbon-like or rod-like) can be controlled by changing the length of methylene chain in the hydrophobic part, the length of methylene chain between the amide and amine oxide groups, and the lengths of methylene chains between amide groups. Furthermore, hydrogels consisting of rod-like aggregates showed significantly higher viscoelasticity than those consisting of ribbon-like aggregates. In other words, it was shown that the gel viscoelasticity could be controlled by changing the methylene chain lengths at four different locations of the AAO.

## 1. Introduction

The control of rheological behavior is critical for a variety of industrial products, such as cosmetics, toiletry, and paints, because rheological properties are closely related to product characteristics, dispersion and emulsion stability, and the feel of the product. Although polymer materials are commonly used as thickeners/gelators, they have significant disadvantages: their molecular weight is difficult to control, and once the polymer is dissolved, the viscosity of the gel does not decrease even at high temperatures, resulting in poor operability. In contrast to conventional polymer gels, supramolecular gels have attracted attention in recent years [1,2,3,4,5,6,7,8,9,10,11,12,13,14,15,16,17,18,19,20,21,22,23,24,25,26,27,28,29,30]. Supramolecular gels are formed by the self-assembly of low-molecular-weight gelators (LMWGs), which form fibrous aggregates and 3D network structures with them. Compared to polymer gelators, LMWGs are easier to synthesize, and above the gelation temperature, they do not significantly increase the viscosity, making them easier to handle. Another advantage of LMWGs is that their gelation temperature can be controlled by their chemical structure. At the same time, since self-assembly is based on intermolecular interactions, such as solvophobic interactions, van der Waals interactions, hydrogen bonds, π–π interactions, metal coordination, and host–guest interaction, supramolecular gels (LMWG gels) have lower strength than polymer gels, in which monomers are covalently bonded. Furthermore, because the thickening and gelation performance of LMWGs is extremely sensitive to their chemical structure, few guidelines for the design of gelators for industrial applications have been reported; thus, the development of LMWGs has required time and resources.

We have previously reported that amidoamine oxide surfactants (AAOs), which are long-chain alkylamine oxides with multiple amide groups, gelate water and aqueous salt solutions [29,30,31]. Long-chain alkylamine oxides are general-purpose surfactants used in kitchen detergents. Multiple amide groups are introduced for the formation of hydrogen bonds between neighboring AAO molecules. Although AAOs are achiral molecules, they have sufficient thickening and gelation ability in polar solvents. AAOs also possess notable industrial advantages: they are easy to synthesize and purify, and their production cost is low. In addition, the gelation temperatures and rheological properties of AAOs may be adjusted through slight changes in molecular structure, in particular, in the length of the alkyl chain in the hydrophobic part, number and arrangement of amide groups, and the length of the methylene chain between the amide and amine oxide groups. The objective of this study was to clarify the relationship between the chemical structure of AAOs, their aggregate structure, and rheological behavior, and to formulate guidelines for the design of LMWGs. In this paper, we report the results of our investigation into the effects of the length of the methylene chain in AAOs on the aggregate structure and rheological properties of their hydrogels.

## 2. Results and Discussion

**Figure 1:**
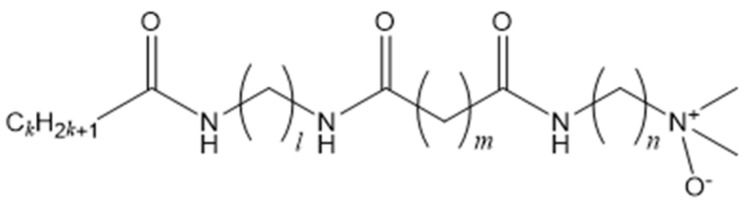
Chemical structure of alkyl amidoamine oxide. *k* is the length of the methylene chain of the hydrophobic part, *l* is the length of the methylene chain between nitrogen atoms of the amide groups, *m* is the length of the methylene chain between carbonyl groups of the amide groups, and *n* is the length of the methylene chain between the amide and amine oxide groups. AAO is denoted as *k*-*l*-*m*-*n* using the length of the methylene chain in four places.

As shown in Figure 1, AAOs have methylene chains at four locations: in the hydrophobic part, between amide groups (between nitrogen atoms and between carbonyl groups), and between amide and amine oxide groups. The effects of the lengths of these methylene chains (*k*, *l*, *m*, and *n*, respectively) on the aggregate structure, *T*_gel_, and rheological behavior were investigated.

**Figure 2:**
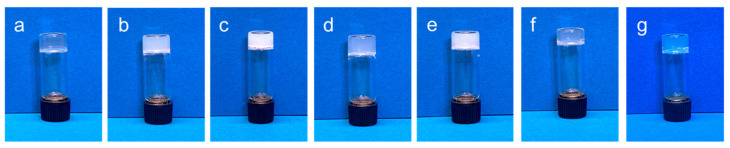
Photographs of hydrogels of certain AAOs at around 25 °C. (**a**) 9-2-2-6, (**b**) 11-2-2-6, (**c**) 13-2-2-6, (**d**) 13-3-2-6, (**e**) 13-4-2-6, (**f**) 13-5-2-6, and (**g**) 13-2-3-6.

Figure 2 shows some examples of the appearance of AAO hydrogels. Depending on the chemical structure, the gel can be transparent or cloudy.

### 2.1. Gelation Temperature (T_gel_)

**Figure 3:**
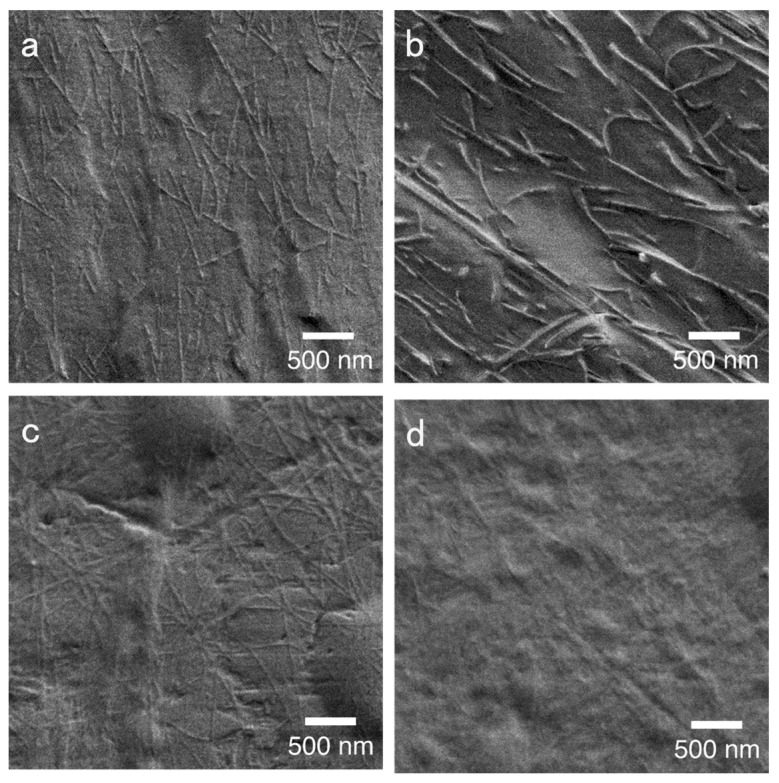
Cryo-SEM images of the surfactant aqueous solutions. Typical images of (**a**) 9-2-2-6 solution quickly frozen from room temperature (<*T*_gel_), (**b**) 11-2-2-6 solution quickly frozen from room temperature (<*T*_gel_), (**c**) 9-2-2-6 solution quickly frozen from about 60 °C (>*T*_gel_), and (**d**) 11-2-2-6 solution quickly frozen from about 80 °C (>*T*_gel_).

The typical cryo-SEM images of aqueous AAO solutions sampled above and below *T*_gel_ are shown in Figure 3. At room temperature, the aqueous AAO solution is highly viscous and does not flow, and the obtained cryo-SEM images (Figure 3a,b) clearly show the presence of aggregates. An aqueous solution of 9-2-2-6 contains thin and straight rod-like structures (Figure 3a), whereas the solution of 11-2-2-6 contains twisted ribbon-like structures (Figure 3b) that are wider than those in 9-2-2-6 [30,31]. Figure 3c shows a quick-frozen aqueous solution of 9-2-2-6 heated to about 60 °C (higher than *T*_gel_), and Figure 3d shows a quick-frozen aqueous solution of 11-2-2-6 heated to about 80 °C (higher than *T*_gel_).

We assumed that these are snapshots of aggregates formed at respective temperatures before freezing. The aqueous solutions of AAOs show low viscosity, almost the same as that of water without AAOs. Despite the low viscosity, aggregates are observed in these solutions at temperatures above *T*_gel_, although in considerably smaller quantities than below *T*_gel_. The AAO concentration *C*_D_ of 50 mM is clearly above the critical micelle concentration; thus, the formation of aggregates above *T*_gel_ is reasonable. However, as the viscosity of the aqueous solution is almost the same as that of the solvent at temperatures above *T*_gel_, the concentration of aggregates with large aggregate numbers is low. Thus, *T*_gel_ is considered to be the temperature at which “aggregates with aggregate number sufficient to induce an increase in viscosity begin to form” through hydrogen bonding between adjacent AAO amide groups.

### 2.2. Effects of the Length of the Methylene Chain between the Amide and Amine Oxide Groups (n)

**Figure 4:**
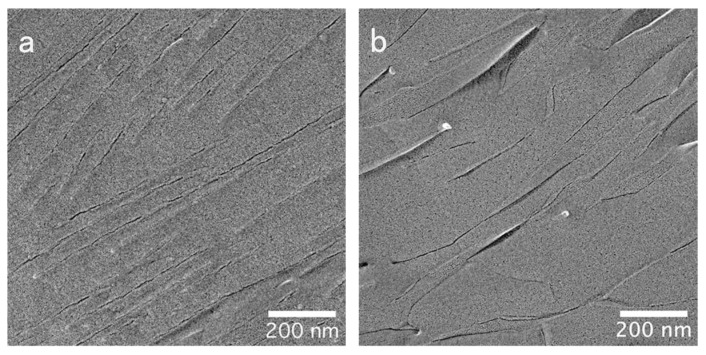
Typical images of 9-2-2-6 (**a**), and 11-2-2-6 (**b**) solutions observed by freeze-fracture TEM.

**Figure 5:**
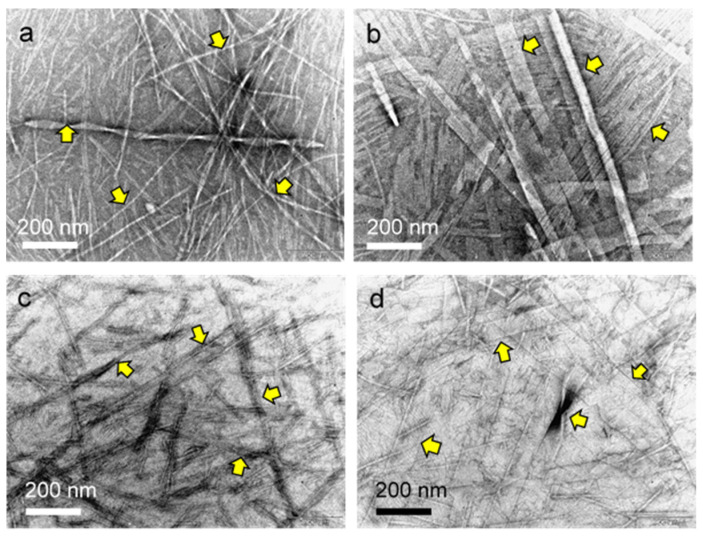
Negative-staining TEM images of the surfactant aqueous solutions at room temperature. Yellow arrows indicate aggregates. Typical images of (**a**) 9-2-2-6 solution, (**b**) 11-2-2-6 solution, (**c**) 13-2-2-4 solution, and (**d**) 13-2-2-6 solution.

We previously reported the relationship between *T*_gel_ and the length of the methylene chain between the amide and amine oxide groups (*n*) in aqueous AAO solutions [30,31]. Briefly, *T*_gel_ increased with *n* for all methylene chain lengths (*k* = 9, 11, and 13 in the hydrophobic part), and cryo-TEM observations showed the formation of thin and linear rod-like aggregates in the region where *T*_gel_ linearly increased with *n* [30,31]. The slope of the plot of *T*_gel_ against *n* (d*T*_gel_/d*n*) was 30, 20, and 15 for *k* = 9, 11, and 13, respectively, indicating that the effect of *n* on *T*_gel_ diminishes with increasing *k*, i.e., with the lengthening of the hydrophobic chain. This was attributed to the decrease in the curvature of the aggregates with increasing *k*. At the same time, the *T*_gel_ vs. *n* curves of both 11-2-2-6 and 13-2-2-6 significantly deviated to the upside from the straight line; notably, a flat ribbon-like structure was observed in these two samples by cryo-TEM [30]. The present cryo-SEM observations (Figure 3) also show an aggregate structure, which is in good agreement with that observed using cryo-TEM. Quick-freeze replica TEM images also reveal thin linear aggregates in 9-2-2-6 (Figure 4a) and wide twisted structures in 11-2-2-6 (Figure 4b). Negative-staining TEM also shows rod-like aggregates in 9-2-2-6 (Figure 5a) and 13-2-2-4 (Figure 5c), and ribbon-like structures in 11-2-2-6 (Figure 5b) and 13-2-2-6 (Figure 5d). In summary, the shapes of the aggregates observed using cryo-TEM, cryo-SEM, quick-freeze replica TEM, and negative-staining TEM are in good agreement with each other. Generally, in negative-staining methods, there are concerns about structural changes in the aggregates caused by staining agents and the drying out of water. However, in this study, the aggregate structures obtained using negative-staining methods are in good agreement with those observed by non-staining cryo-methods, indicating no effect of the staining agent and drying. These results suggest that the relationship between *T*_gel_ and *n* accurately reflects the structure of the aggregates formed in water.

The aggregate structure is closely related to *n* for the following reasons. When *n* is small, the amide groups, which are hydrogen bonding sites, are close to the aggregate surface, i.e., water. In this case, water as a solvent prevents the formation of hydrogen bonds between neighboring molecules. The decrease in the curvature of aggregates due to the increase in *n* also affects the formation of hydrogen bonds.

We previously reported the dependence of cmc on *n* for *N*-lauroylaminoalkyl-*N*′,*N*′-dimethylamine oxide, a molecule consisting of dodecyldimethylamine oxide with one amide group and a methylene chain (chain length *n*) between the amide and amine oxide groups [32]. The cmc hardly changed at 2 ≤ *n* ≤ 4; however, at *n* ≥ 5, the cmc rapidly decreased with increasing *n*. This suggests that when *n* is small, the amide group and the methylene chain between the amide and amine oxide groups form a polar group; at the same time, when *n* is sufficiently large, the methylene chain between the amide and amine oxide groups acts as a hydrophobic chain. This result also supports the relationship between *n* and the aggregate structure described above.

**Figure 6:**
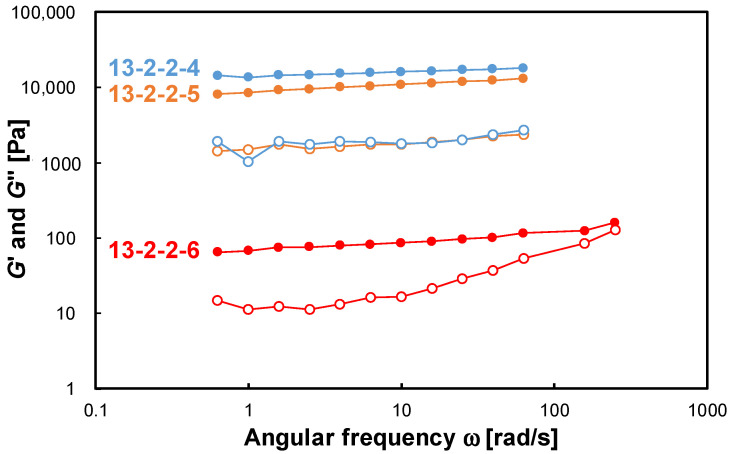
Angular frequency dependence of *G*′ (solid circles) and *G*″ (open circles) of 13-2-2-*n* (*n* = 4, 5, and 6) aqueous solutions at 25 °C.

The differences in the aggregate structure at different *n* were expected to affect the rheology of the hydrogels. The storage modulus, *G*′, and loss modulus, *G*″, of 13-2-2-*n* (*n* = 3–6) hydrogels at 25 °C are shown as functions of angular frequency in Figure 6. Electron micrographs confirm that 13-2-2-4 and 13-2-2-5 form rod-like aggregates and 13-2-2-6 forms ribbon-like aggregates. All gels have *G*′ > *G*″ in the examined angular frequency region, indicating that they are in the gel state. The 13-2-2-4 and 13-2-2-5 hydrogels containing rod-like aggregates show similar viscoelastic behavior. In addition, both *G*′ and *G*″ are almost two orders of magnitude greater for rod-like aggregates than for ribbon-like ones. In other words, hydrogels containing ribbon-like aggregates (more regular or closer to crystalline) are less viscoelastic than those containing rod-like aggregates. The viscoelastic behavior of gels containing ribbon-like aggregates is further discussed. In gels containing ribbon-like aggregates, *G*′ is almost constant regardless of frequency, while *G*″ shows upturns in the curve. An idea presented in organogels may explain this result [15]. That is, given the crystal-like structure of ribbon-like aggregates, gels containing ribbon-like aggregates may form solid networks rather than physical cross-links.

### 2.3. Effects of the Length of Methylene Chain in the Hydrophobic Part (k)

**Figure 7:**
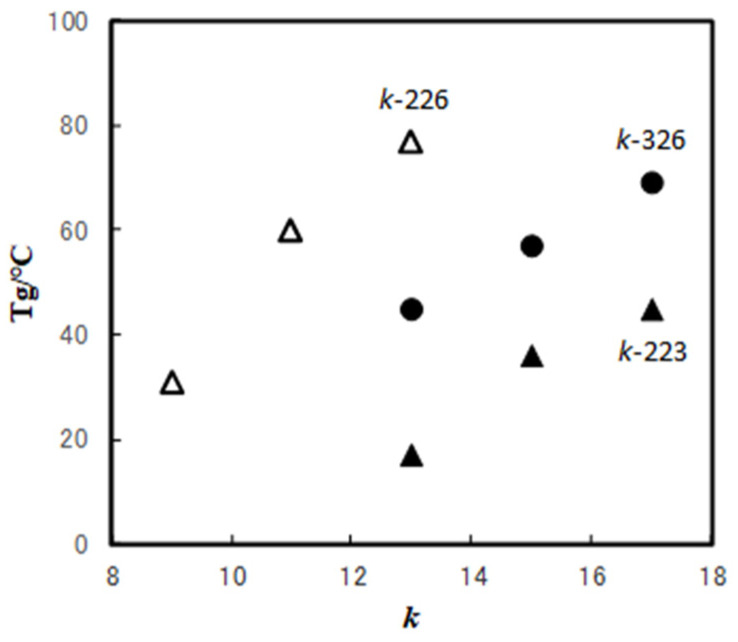
The *k*-dependence of the *T*_gel_ of *k*-2-2-3 (solid triangles), *k*-2-2-6 (open triangles) [31], and *k*-3-2-6 (solid circles).

The effect of the length of the methylene chain in the hydrophobic part (*k*) on *T*_gel_, aggregate structures, and viscoelasticity are discussed. The value of *k* affects the hydrophobic interaction between AAO molecules in water. The relationship between *T*_gel_ and *k* is shown in Figure 7: *T*_gel_ increases with *k* in all cases [31]. The slopes (d*T*_gel_/d*k*) for *k*-2-2-3 and *k*-3-2-6, which formed rod-like aggregates, are 7.0 and 6.0, respectively. At the same time, for *k*-2-2-6 (11 ≤ *k* ≤ 13), which formed ribbon-like aggregates, the slope is 17, which is considerably higher than that for the rod-like aggregates. Ribbon-like aggregates have a lower curvature and thus a shorter distance between neighboring molecules than rod-like aggregates, suggesting a greater contribution of intermolecular hydrogen bonds to the stabilization of the former aggregates. At *k* = 13, the *T*_gel_ values for 13-2-2-6, 13-3-2-6, and 13-2-2-3 are 77, 45, and 17 °C, respectively. Although 13-3-2-6 and 13-2-2-3 both form similar rod-like aggregates, their *T*_gel_ values differ by 28 °C. In other words, even aggregates with similar rod-like structures have significantly different temperature stabilities owing to differences in the lengths of methylene chains other than the hydrophobic chain.

**Figure 8:**
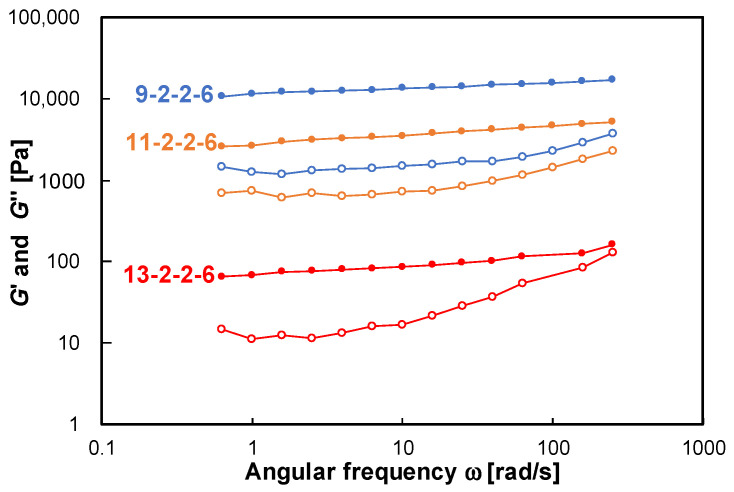
Angular frequency dependence of *G*′ (solid circles) and *G*″ (open circles) of the *k*-2-2-6 (*k* = 9, 11, and 13) aqueous solutions at 25 °C.

Next, the relationships of *G*′ and *G*″ with an angular frequency of *k*-2-2-6 (*k* = 9, 11, and 13) were investigated, and the results are shown in Figure 8. In all the examined regions, *G*′ is larger than *G*″ in the gelled state. Both *G*′ and *G*″ are larger when *k* is small. As mentioned above, an aqueous solution of 9-2-2-6 was confirmed to have rod-like aggregates, whereas the aqueous solutions of 11-2-2-6 and 13-2-2-6 have ribbon-like aggregates. Ribbon-like aggregates have a more regular arrangement of molecules, such as in solid or crystalline structures, whereas rod-like aggregates have a larger curvature and greater intermolecular distances than ribbon-like aggregates. Hydrogels with ribbon-like aggregates are less viscous and less elastic than gels with rod-like aggregates. In the present study, similar results were obtained for samples with different *n*, indicating that differences in the aggregate structure affect the viscoelasticity of the hydrogels.

### 2.4. Effects of the Lengths of Methylene Chains between Amide Groups (l and m)

**Figure 9:**
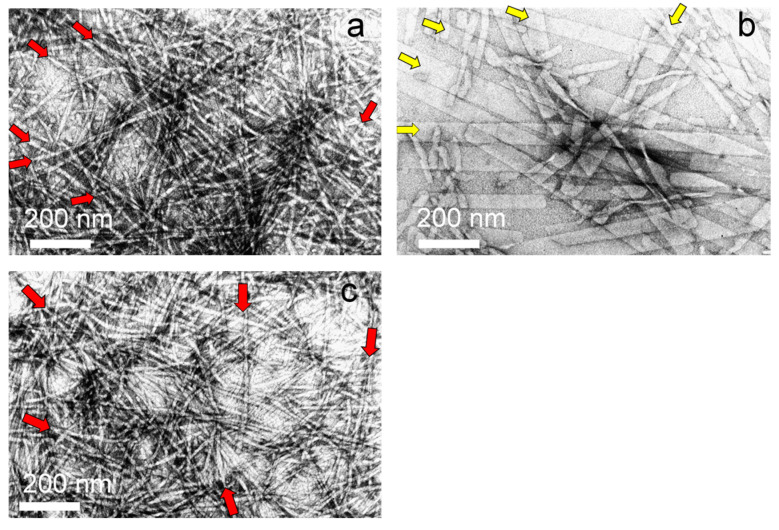
Negative-staining TEM images of the surfactant aqueous solutions at room temperature. Red arrows indicate rod-like aggregates, and yellow arrows indicate ribbon-like aggregates. Typical images of (**a**) 13-3-2-6 solution, (**b**) 13-4-2-6 solution, and (**c**) 13-5-2-6 solution.

**Figure 10:**
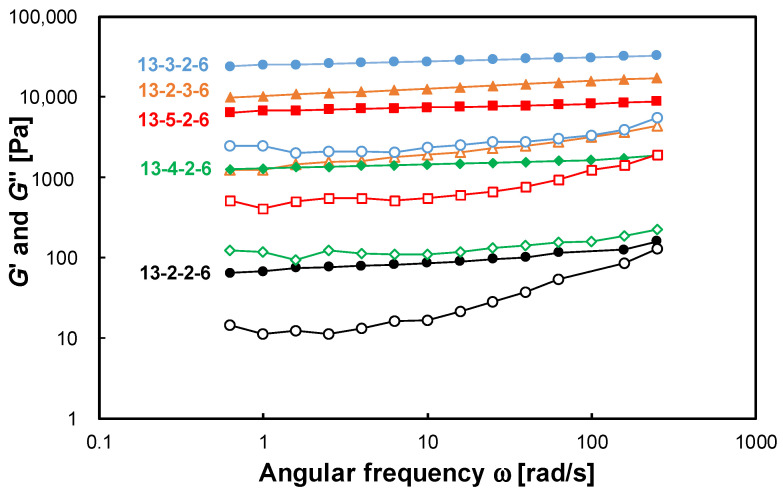
Angular frequency dependence of *G*′ (solid symbols) and *G*″ (open symbols) of 13-*l*-*m*-6 (*l* = 2–5; *m* = 2 and 3) aqueous solutions at 25 °C.

The aggregate structure and rheological behavior were examined for different lengths of the methylene chains between the amide groups (*l* and *m*). The values of *T*_gel_ are listed in Table 1. Interestingly, when either *l* or *m* is odd, the *T*_gel_ is much lower than when both are even [31]. The odd–even effect of methylene chain length was not observed for the hydrophobic part or the length of the methylene chain between the amide and amine oxide groups, suggesting that the role of methylene chains between the amide groups differs from that of the other methylene chains. The negative-staining TEM images of the formed aggregates are shown in Figure 9. There are significant differences in the aggregate structures, with rod-like or bundle-like structures (Figure 9a,c) observed for odd lengths of the methylene chain between amide groups and wide, flat ribbon-like structures (Figure 9b) observed for methylene chains of even length. The investigation of the rheological properties of these samples (Figure 10) shows that both *G*′ and *G*″ are larger when either *l* or *m* is odd than when both *l* and *m* are even. In other words, hydrogels with rod-like, or bundle-like aggregates show higher viscoelasticity than those with ribbon-like aggregates.

**Figure 11:**
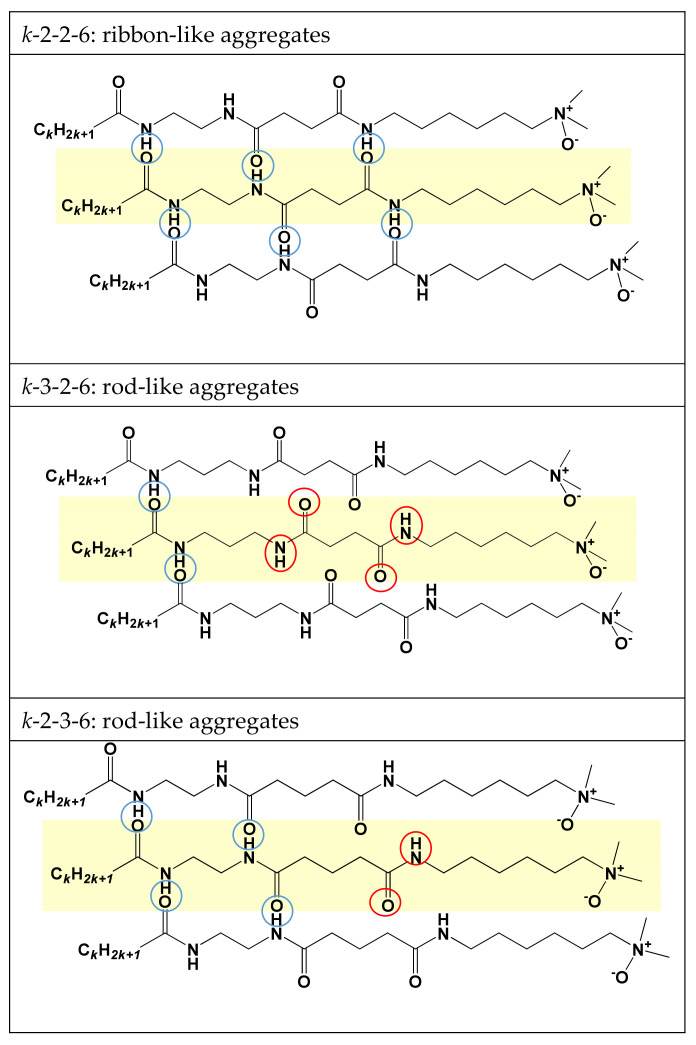
Schematic of the odd–even effect of hydrogen bonding formation. Blue circles indicate hydrogen bonds between neighboring AAO molecules. Red circles indicate amide groups without hydrogen bonds.

The odd–even effect of the lengths of methylene chains between amide groups on *T*_gel_ was attributed to the number of hydrogen bonding points formed between amide groups of the neighboring molecules. As reported by Sumiyoshi et al. [33,34], the numbers of hydrogen bonds formed between adjacent molecules are different for odd and even lengths of the methylene chains between amide groups, as shown in Figure 11. This effect is similar to the parallel–antiparallel model for the β-sheet of polypeptides such as proteins; it also explains why *T*_gel_ increases, more lamellar and flat aggregate structures are formed, and the curvature of aggregates decreases (forming rod-like to ribbon-like aggregates) with an increasing number of hydrogen bonds. At the same time, the bundle structure, which is an aggregation of thin rod-like aggregates, shows better viscoelasticity than the ribbon-like structure. Electron micrographs show that the widths of ribbon-like aggregates are tens of nm, whereas the diameters of the rod-like aggregates are a few nm; furthermore, the numbers of aggregates are significantly different. In other words, at the same AAO concentration, the number of rod-like aggregates is much greater than that of ribbon-like aggregates. Therefore, the rod-like aggregates have a larger number of mutual entanglement points, and as a result, they exhibit higher viscoelasticity.

## 3. Conclusions

In this paper, we reported the relationship between the lengths of methylene chains at four different locations in AAO and the aggregate structure, *T*_gel_, and viscoelasticity of the hydrogel. A significant odd–even effect on *T*_gel_ was observed for the lengths of the methylene chains between the amide groups (*l* and *m*). At the same time, *T*_gel_ monotonically increased with the lengths of the methylene chains in the hydrophobic part (*k*) and between the amide and amine oxide groups (*n*), and no odd–even effect was observed for *k* and *n*. This difference suggests that each methylene chain plays a different role in aggregate formation. Specifically, *k* and *n* determine the curvature of the aggregates, whereas *l* and *m* directly affect the number of intermolecular hydrogen bonds.

The aggregate structures were observed using various microscopic techniques, including cryo-TEM, cryo-SEM, quick-freeze replica TEM, and negative-staining TEM. Our results confirmed that the aggregate structure was almost the same in this system, and the staining agent hardly changed the aggregate structure.

Furthermore, we found that the ribbon-like aggregates are formed only when all the following conditions are met: *k ≥* 11, *n ≥* 6, and *l* and *m* are both even. If even one of these conditions is not met, the aggregates have a rod-like structure. At the same time, hydrogels with rod-like aggregates showed higher viscoelasticity than those with ribbon-like aggregate, and a strong correlation was observed between the aggregate structure and viscoelasticity. In other words, we succeeded in determining the parameters of the chemical structure of the AAO that control the *T*_gel_ and rheological behavior.

There is a high demand for gelators that can be synthesized cost-effectively for industrial applications. The molecular design guidelines obtained in this study can be applied not only to hydrogelators but also to organogelators.

## 4. Materials and Methods

### 4.1. Materials

**Scheme 1:**
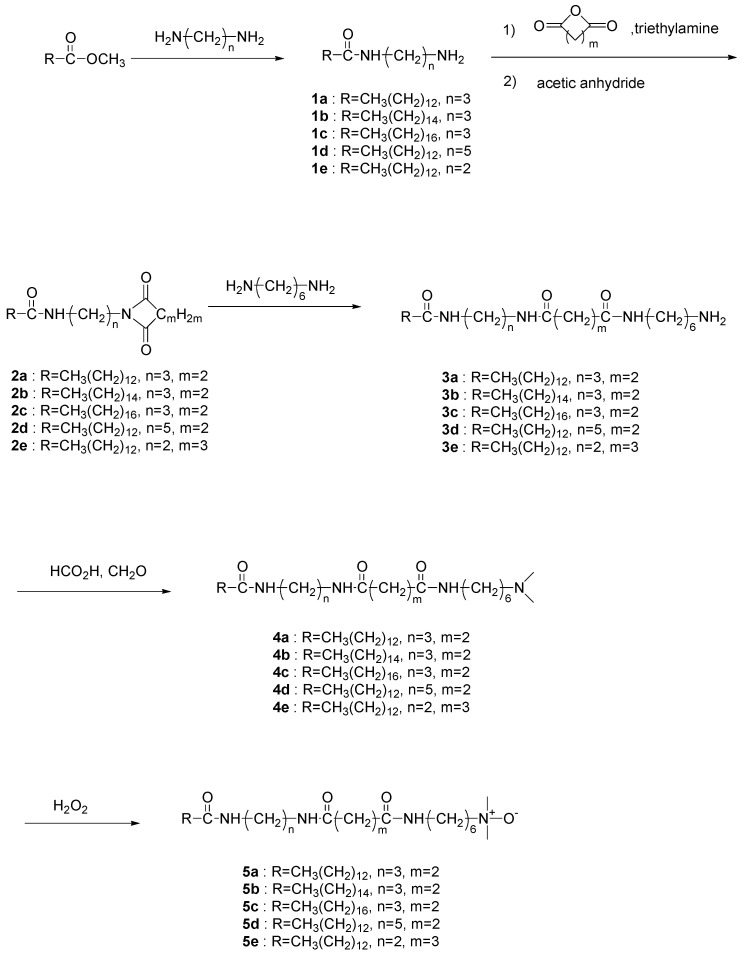
Synthesis of alkyl amidoamine oxides used in this study.

The general synthetic routes of aminoxides are shown in Scheme 1. 1,3-Diaminopropane, 1,5-diaminopentane, succinic anhydride, glutaric anhydride, hexamethylenediamine, solvents, and other reagents were of commercial grade and were not additionally purified before use.

#### 4.1.1. Synthesis of *N*-acylethlalkyldiamine (**1a**, **1b**, **1c**, **1d**, **1e**): General Procedure

Methyl myristate (35.0 g, 144 mmol) and 1,3-diaminopropane (32.1 g, 433 mmol) were stirred at 120 °C for 48 h. The reaction mixture was poured into methanol and filtered. The filtrate was evaporated, and the resulting residue was purified by recrystallization in hexane. *N*-myristoylpropanediamine (**1a**) was obtained with 60% yield (24.6 g, 86.6 mmol) as a white crystalline powder.

#### 4.1.2. Synthesis of *N*-acylaminoalkylsuccinimide (**2a**, **2b**, **2c**, **2d**): General Procedure

*N*,*N*-dimethylformamide (60 mL) solution containing *N*-myristoylpropanediamine (**1a**) (15.0 g, 52.7 mmol) and trimethylamine (10.7 g, 105 mmol) was added to solid succinic anhydride (5.54 g, 55.4 mmol) for 10 min and stirred for 30 min at 70 °C. After succinic anhydride was completely dissolved, acetic anhydride (8.07 g, 79.1 mmol) was added dropwise for 10 min to the reaction solution, followed by stirring at 100 °C for 1 h. The reaction mixture was poured into water (200 mL), and the precipitate was filtered and washed with water. The precipitate was purified by recrystallization in methanol. *N*-myristoylaminopropylsuccinimide (**2a**) was obtained with 92% yield (17.7 g, 48.5 mmol) as a white crystalline powder.

#### 4.1.3. Synthesis of N-myristoyletylglutarimide (**2e**)

*N*,*N*-dimethylformamide (100 mL) solution containing *N*-myristoyletylenediamine (**1e**) (12.0 g, 44.4 mmol) and trimethylamine (8.98 g, 88.7 mmol) was added to glutaric anhydride (5.82 g, 51.0 mmol) for 10 min and stirred for 30 min at 70 °C. After glutaric anhydride completely dissolved, the reaction solution was added dropwise for 10 min to acetic anhydride (6.79 g, 66.6 mmol) and stirred at 100 °C for 3 h. The reaction mixture was poured into water (200 mL), and the precipitate was filtered and washed with water. The precipitate was purified by recrystallization in methanol. *N*-myristoylaminoetylglutarimide (**2e**) was obtained with 76% yield (12.3 g, 33.7 mmol) as a white crystalline powder.

#### 4.1.4. Synthesis of *N*-(acylaminoalkyl)succinamoylaminohexylamine (**3a**, **3b**, **3c**, **3d**) and [(*N*-myristoyl)glutaramoylaminohexyl]dimethylamine (**3e**): General Procedure

*N*-myristoylaminopropylsuccinimide (**2a**) (12.0 g, 32.7 mmol) and hexamethylenediamine (15.2 g, 131 mmol) were mixed and stirred at 120 °C for 18 h. The reaction mixture was poured into methanol, and the precipitate was filtered and washed with methanol. The resulting solid was purified by recrystallization in methanol. *N*-(myristoylaminopropyl)succinamoylaminohexylamine (**3a**) was obtained with 62% yield (9.80 g, 20.3 mmol) as a white crystalline powder.

#### 4.1.5. Synthesis of [(*N*-acylaminoalkyl)succinamoylaminohexyl]dimethylamine (**4a**, **4b**, **4c**, **4d**) and [(*N*-myristoyl)glutaramoylaminohexyl]dimethylamine (**4e**): General Procedure

*N*-(myristoylaminopropyl)succinamoylaminohexylamine (**3a**) (8.0 g, 16.6 mmol), 37% formaldehyde (80.9 mL), and formic acid (4.58 g, 99.6 mmol) were added to 2-propanol (20 mL) and stirred at 90 °C for 4 h. The reaction mixture was poured into 2N sodium carbonate (150 mL) and extracted with chloroform (100 mL) twice. After the evaporation of solvents and purification by column chromatography (silica gel, chloroform:methanol = 1:2), [(*N*-myristoylaminopropyl)succinamoylaminohexyl]dimethylamine (**4a**) was obtained with 92% yield (7.79 g, 15.2 mmol) as a white solid.

#### 4.1.6. Synthesis of [(*N*-acylaminoalkyl)succinamoylaminohexyl]dimethylamine oxide (**5a**, **5b**, **5c**, **5d**) and [(*N*-myristoylaminoetyl)glutaramoylaminohexyl]dimethylamine oxide (**5e**): General Procedure

[(*N*-myristoylaminopropyl)succinamoylaminohexyl]dimethylamine (**4a**) (7.5 g, 14.7 mmol) solution in 2-propanol (20 mL) and 30% hydrogen peroxide (6.26 g, 58.7 mmol) were mixed and stirred at 50 °C for 4 h. Next, Pd/C (20 mg) was added to the solution, followed by stirring for 24 h. The solution was filtered, and the filtrate was evaporated. The residue was purified by column chromatography (silica gel, chloroform: methanol = 1:2) and recrystallized in acetone/methanol. [(*N*-myristoylaminopropyl)succinamoylaminohexyl]dimethylamine oxide (**5a**) was obtained with 49% yield (3.79 g, 7.20 mmol) as a white crystalline powder.

The AAO concentrations of all aqueous solutions were 50 mM. In addition, no pH adjustments were performed.

### 4.2. Characterization

^1^H NMR spectra were recorded with a JEOL ECZ400 (400 MHz) spectrometer (Tokyo, Japan). These spectra are shown in Figures S1–S10.

9-2-2-6: ^1^H NMR (δ ppm, CD_3_OD, 297K, 400 MHz): 0.90 (t, 3H, J = 6.8), 1.22–1.46 (m, 16H), 1.46–1.64 (m, 4H), 1.79–1.90 (m, 2H), 2.18 (t, 2H, J = 7.6), 2.40–2.50 (m, 4H), 3.14 (s, 6H), 3.21–3.35 (m, 8H).

11-2-2-6: ^1^H NMR (δ ppm, CD_3_OD, 297K, 400 MHz): 0.90 (t, 3H, J = 6.8), 1.22–1.48 (m, 20H), 1.52–1.64 (m, 2H), 1.79–1.88 (m, 2H), 2.18 (t, 2H, J = 7.4), 2.42–2.50 (m, 4H), 3.17 (s, 6H), 3.21–3.36 (m, 8H).

13-2-2-3: ^1^H NMR (δ ppm, CD_3_OD, 297K, 400 MHz): 0.90 (t, 3H, J = 6.8), 1.22–1.39 (m, 20H), 1.46–1.64 (m, 2H), 1.98–2.07 (m, 2H), 2.17 (t, 2H, J = 7.6), 2.42–2.52 (m, 4H), 3.14 (s, 6H), 3.21–3.33 (m, 8H).

13-2-2-4: ^1^H NMR (δ ppm, CD_3_OD, 297K, 400 MHz): 0.90 (t, 3H, J = 6.8), 1.22–1.38 (m, 20H), 1.50–1.63 (m, 4H), 1.82–1.91 (m, 2H), 2.18 (t, 2H, J = 7.4), 2.40–2.50 (m, 4H), 3.16 (s, 6H), 3.21–3.33 (m, 8H).

13-2-2-5: ^1^H NMR (δ ppm, CD_3_OD, 297K, 400 MHz): 0.90 (t, 3H, J = 6.8), 1.23–1.44 (m, 22H), 1.52–1.64 (m, 4H), 1.82–1.91 (m, 2H), 2.18 (t, 2H, J = 7.6), 2.40–2.49 (m, 4H), 3.15 (s, 6H), 3.21–3.33 (m, 8H).

13-2-2-6: ^1^H NMR (δ ppm, CD_3_OD, 297K, 400 MHz): 0.90 (t, 3H, J = 6.8), 1.22–1.46 (m, 24H), 1.48–1.64 (m, 4H), 1.79–1.89 (m, 2H), 2.18 (t, 2H, J = 7.6), 2.40–2.49 (m, 4H), 3.14 (s, 6H), 3.22–3.33 (m, 8H).

13-3-2-6: ^1^H NMR (δ ppm, CD_3_OD, 297K, 400 MHz): 0.90 (t, 3H, J = 6.8), 1.22–1.46 (m, 24H), 1.48–1.72 (m, 6H), 1.79–1.90 (m, 2H), 2.17 (t, 2H, J = 7.6), 2.42–2.49 (m, 4H), 3.14 (s, 6H), 3.22–3.33 (m, 8H).

13-4-2-6: ^1^H NMR (δ ppm, CD_3_OD, 297K, 400 MHz): 0.90 (t, 3H, J = 6.8), 1.22–1.46 (m, 24H), 1.46–1.63 (m, 8H), 1.79–1.88 (m, 2H), 2.16 (t, 2H, J = 7.6), 2.41–2.49 (m, 4H), 3.14 (s, 6H), 3.23–3.33 (m, 8H).

13-5-2-6: ^1^H NMR (δ ppm, CD_3_OD, 297K, 400 MHz): 0.90 (t, 3H, J = 6.8), 1.22–1.45 (m, 26H), 1.46–1.63 (m, 8H), 1.80–1.88 (m, 2H), 2.16 (t, 2H, J = 7.6), 2.41–2.49 (m, 4H), 3.14 (s, 6H), 3.23–3.33 (m, 8H).

13-2-3-6: ^1^H NMR (δ ppm, CD_3_OD, 297K, 400 MHz): 0.90 (t, 3H, J = 6.8), 1.22–1.45 (m, 24H), 1.46–1.63 (m, 4H), 1.80–1.92 (m, 4H), 2.14–2.23 (m, 4H), 2.41–2.49 (m, 4H), 3.14 (s, 6H), 3.22–3.33 (m, 8H).

Accurate mass spectra were acquired using an X500R mass spectrometer (SCIEX, Framingham, MA, USA) running in positive-ion electrospray mode. The ionspray voltage was 5500 V. The nebulizer and heater gas were set at 60 psi and 40 psi, respectively. The resolving power of the mass spectrometer was over 20,000. Each 0.1 μg/mL methanolic solution was infused at a flow rate of 10 μL/min using a syringe pump, YSP-201 (YMC, Kyoto, Japan). Mass spectra in the range of *m*/*z* 100 to 1100 were acquired for 1 min and averaged. ESI mass spectra are shown in Figure S11.

### 4.3. Viscosity Measurement

Using the tuning fork vibro viscometer SV10A (A&D, Tokyo, Japan), the aqueous AAO solution was first heated to the temperature at which the solution viscosity was comparable to that of the solvent, and then, the temperature and viscosity were measured simultaneously during slow cooling. The temperature at which the viscosity rapidly increased with decreasing temperature was defined as the gelation temperature *T*_gel_.

### 4.4. Rheological Measurement

The viscoelasticity of AAO hydrogels was measured using an MCR702 rheometer (Anton Paar Japan, Tokyo, Japan) with a 50 mm diameter parallel-plate geometry. Peltier system was employed for temperature control. A solvent trap was used to reduce evaporation. After the temperature of the aqueous AAO sample was sufficiently higher than *T*_gel_, the sample was introduced into the apparatus. For every sample, frequency, and amplitude sweeps were performed to determine the linear viscoelastic regime. Amplitude sweeps were performed at a fixed frequency of 1 Hz. All rheological measurements were conducted at 25 °C.

### 4.5. Electron Microscopy

#### 4.5.1. Quick-Freezing of Surfactant Aqueous Solutions

Surfactant aqueous solutions were quick-frozen via the metal-contact method using a quick-freeze unit (Polaron E7200, Watford, UK). The surface of a pure copper block was mirror-finished beforehand for optimal thermal conductivity. A drop of each sample with a volume of several microliters was quick-frozen via contact with the surface of the block pre-cooled with liquid helium. The frozen samples were stored in liquid nitrogen for subsequent cryo-SEM and freeze-fracture replication.

#### 4.5.2. Cryo-Scanning Electron Microscopy (Cryo-SEM)

Cryo-SEM was performed using Zeiss LEO 1530 (Oberkochen, Germany) equipped with a cryo-preparation system (Gatan Alto 2500, Pleasanton, CA, USA). The quick-frozen sample was mounted on the sample stage in the cryo-preparation system under vacuum at about 10^−4^ Pa, fractured at −100 °C, sputter-coated with platinum, and transferred to the sample stage for observation at 20 kV.

#### 4.5.3. Freeze-Fracture Replica Transmission Electron Microscopy (TEM)

Freeze-replica films of surfactant aqueous solutions were prepared using a freeze-replica apparatus (BAF 400D, Balzers, Liechtenstein). Quick-frozen sample was mounted on the sample stage in the apparatus at −100 °C under vacuum at about 10^−5^ Pa, fractured, and coated with a layer of platinum (thickness of about 6.5 nm) by evaporation at an angle of 25° while the sample stage was rotating horizontally (low-angle rotary shadowing). The sample was further coated with a carbon protective film (thickness of about 25 nm) by evaporation from an angle of 90°. The replica films were sequentially washed with a kitchen bleach solution, pure water, dilute sulfuric acid, and pure water and were mounted on a TEM copper grid (Veco, 150 mesh, Eerbeek, The Netherlands) in air at room temperature. The specimens were observed using a Philips CM200UT (Eindhoven, The Netherlands) operating at 200 kV.

#### 4.5.4. Negative-Staining TEM

Carbon-coated copper grids (GLF-C10, Okenshoji Co., Ltd., Tokyo, Japan) were used for TEM. A drop of hydrogel sample was placed on a grid and stained with an aqueous solution containing uranyl acetate (2.0 wt%). Sample-loaded grids were vacuum-dried and observed using a JEM-2100 transmission electron microscope (JEOL Ltd., Tokyo, Japan) at an operating voltage of 100 kV.

## Figures and Tables

**Table 1:** Relationship between the chemical structure of AAO and *T*_gel_.

*k*	*l*	*m*	*n*	*T*_gel_/°C
13	2	2	6	77 *
13	2	3	6	53
13	3	2	6	45 *
13	4	2	6	83 *
13	5	2	6	59 *
15	2	2	6	>80
15	3	2	6	57
17	2	2	6	>80
17	3	2	6	69

* ref. [31].

## Data Availability

Not applicable.

## Supplementary Materials

The following supporting information can be downloaded at: https://www.mdpi.com/article/10.3390/gels9030261/s1, Figure S1: ^1^H NMR spectrum of 9-2-2-6; Figure S2: ^1^H NMR spectrum of 11-2-2-6; Figure S3: ^1^H NMR spectrum of 13-2-2-3; Figure S4: ^1^H NMR spectrum of 13-2-2-4; Figure S5: ^1^H NMR spectrum of 13-2-2-5; Figure S6: ^1^H NMR spectrum of 13-2-2-6; Figure S7: ^1^H NMR spectrum of 13-3-2-6; Figure S8: ^1^H NMR spectrum of 13-4-2-6; Figure S9: ^1^H NMR spectrum of 13-5-2-6; Figure S10: ^1^H NMR spectrum of 13-2-3-6; Figure S11: Mass spectrometry of AAOs we synthesized in this study.
