# Supplementary material for: Amidoamine Oxide Surfactants as Low-Molecular-Weight Hydrogelators: Effect of Methylene Chain Length on Aggregate Structure and Rheological Behavior
Source: Gels. 2023 Mar 22;9(3):261. doi: 10.3390/gels9030261 (PMC10048289; doi:10.3390/gels9030261)
Supplement: Supplementary file 1 [file gels-09-00261-s001.zip › Figure S11_revised.pdf]

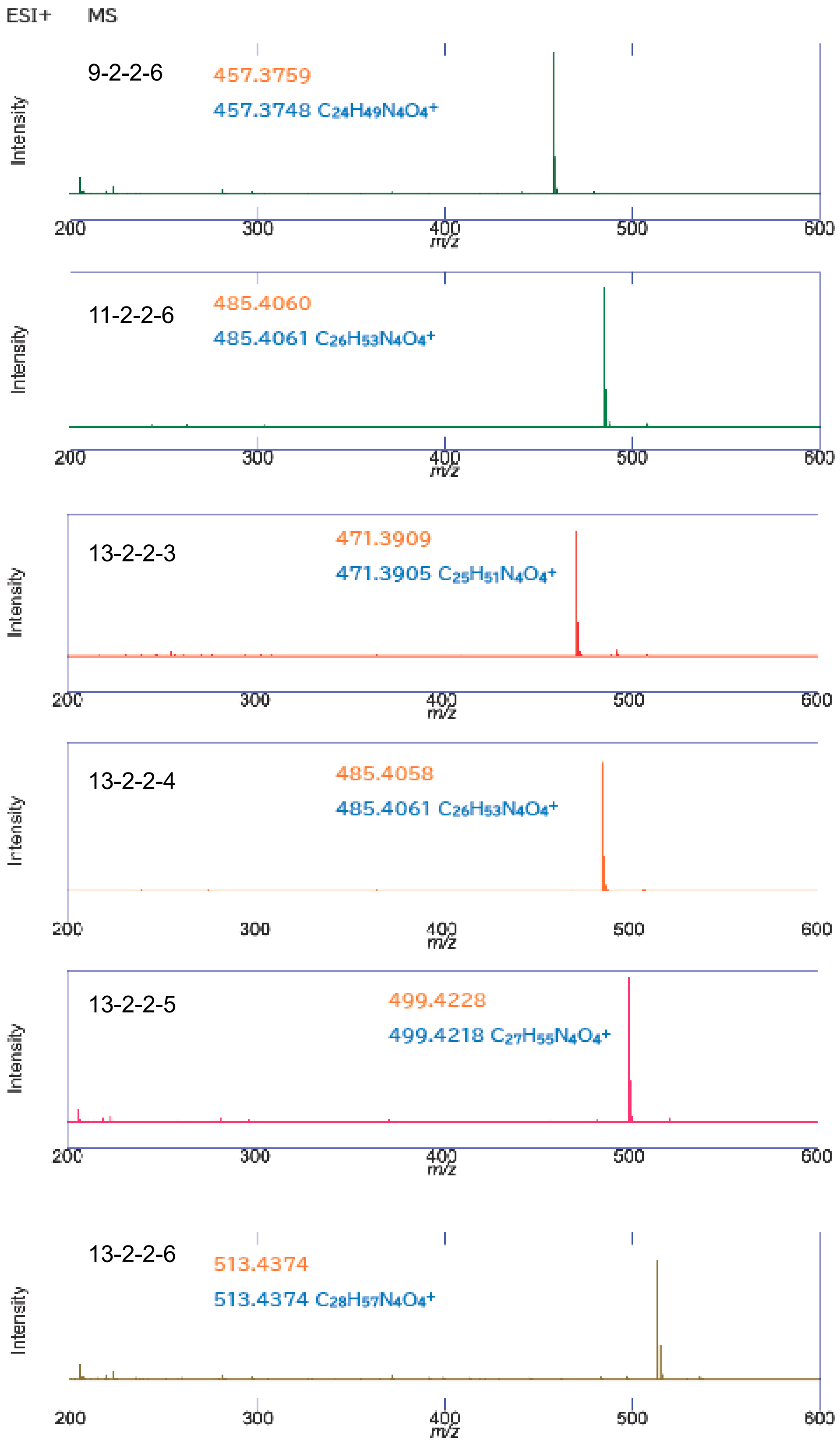
ESI+ MS
9-2-2-6
457.3759
457.3748 $C_{24}H_{49}N_4O_4^+$
11-2-2-6
485.4060
485.4061 $C_{26}H_{53}N_4O_4^+$
13-2-2-3
471.3909
471.3905 $C_{25}H_{51}N_4O_4^+$
13-2-2-4
485.4058
485.4061 $C_{26}H_{53}N_4O_4^+$
13-2-2-5
499.4228
499.4218 $C_{27}H_{55}N_4O_4^+$
13-2-2-6
513.4374
513.4374 $C_{28}H_{57}N_4O_4^+$
Intensity
m/z
200
300
400
500
600

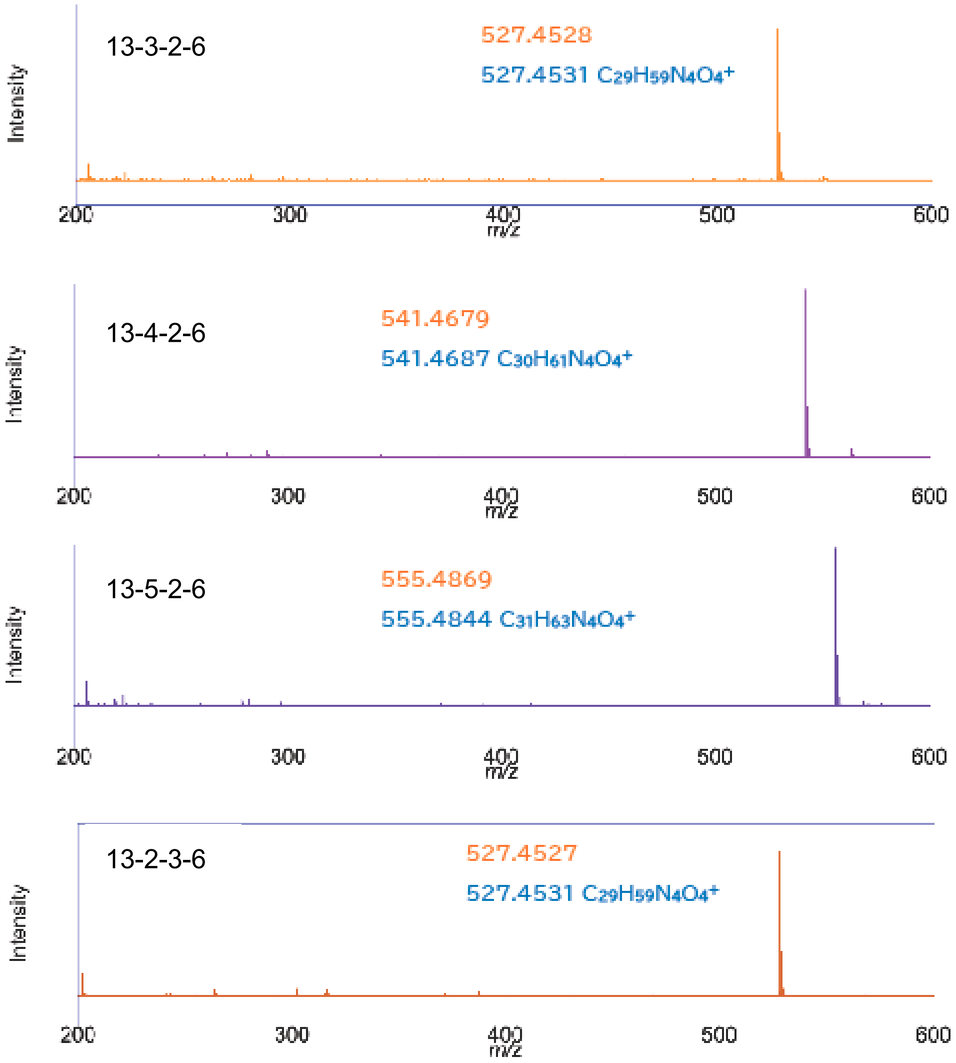

Figure S11. Mass spectrometry of AAOs we synthesized in this study. In the figure, the values in the upper row are actual measured values, and the values in the lower row are calculated values from the molecular formula listed.
