# Supplementary material for: Amidoamine Oxide Surfactants as Low-Molecular-Weight Hydrogelators: Effect of Methylene Chain Length on Aggregate Structure and Rheological Behavior
Source: Gels. 2023 Mar 22;9(3):261. doi: 10.3390/gels9030261 (PMC10048289; doi:10.3390/gels9030261)
Supplement: Supplementary file 1 [file gels-09-00261-s001.zip › Figures S1-10_NMR chart_revised.pdf]

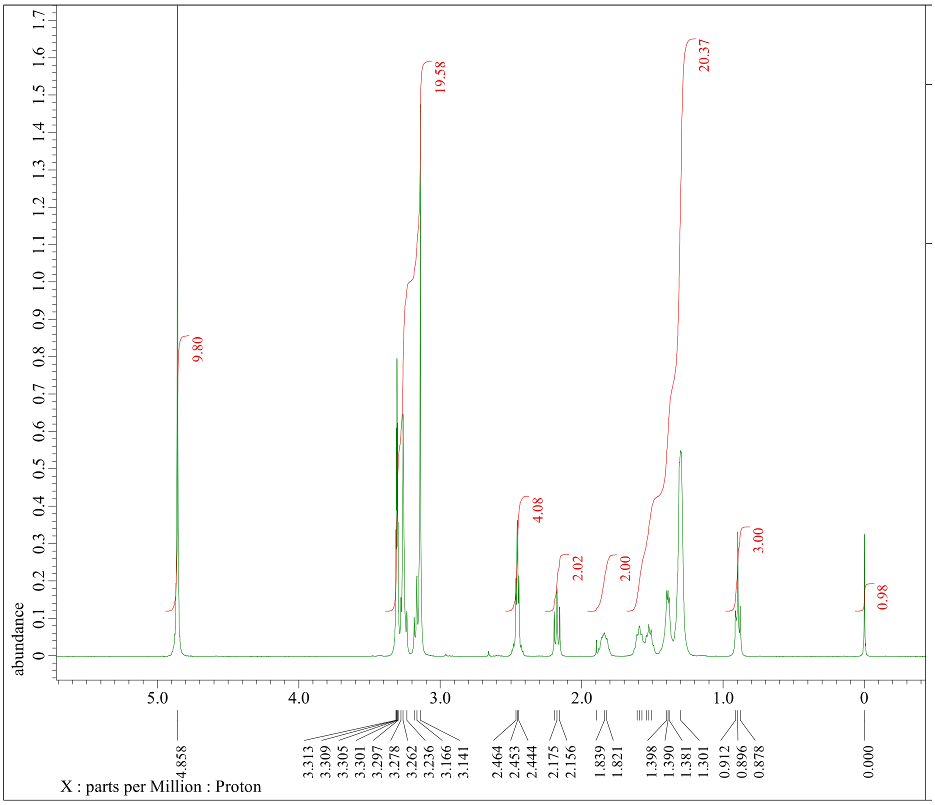

Figure S1. ^{1}H NMR spectrum of 9-2-2-6.

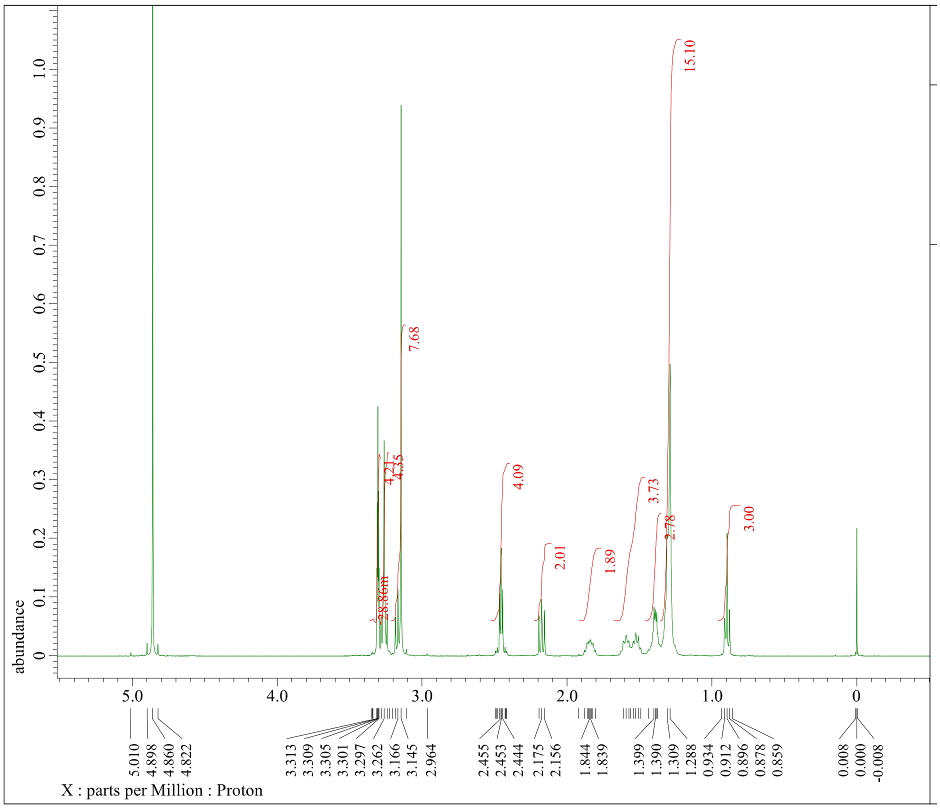

Figure S2. ^{1}H NMR spectrum of 11-2-2-6.

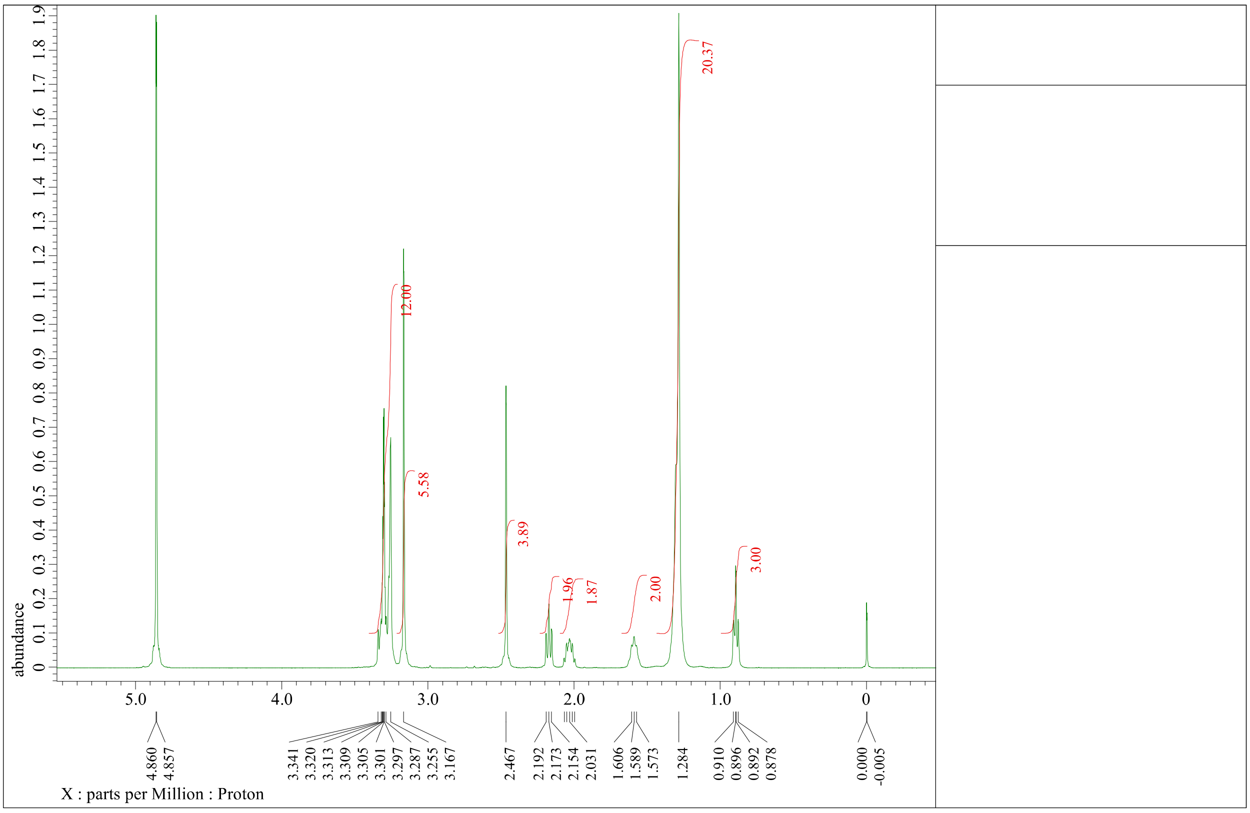

Figure S3. ¹H NMR spectrum of 13-2-2-3.

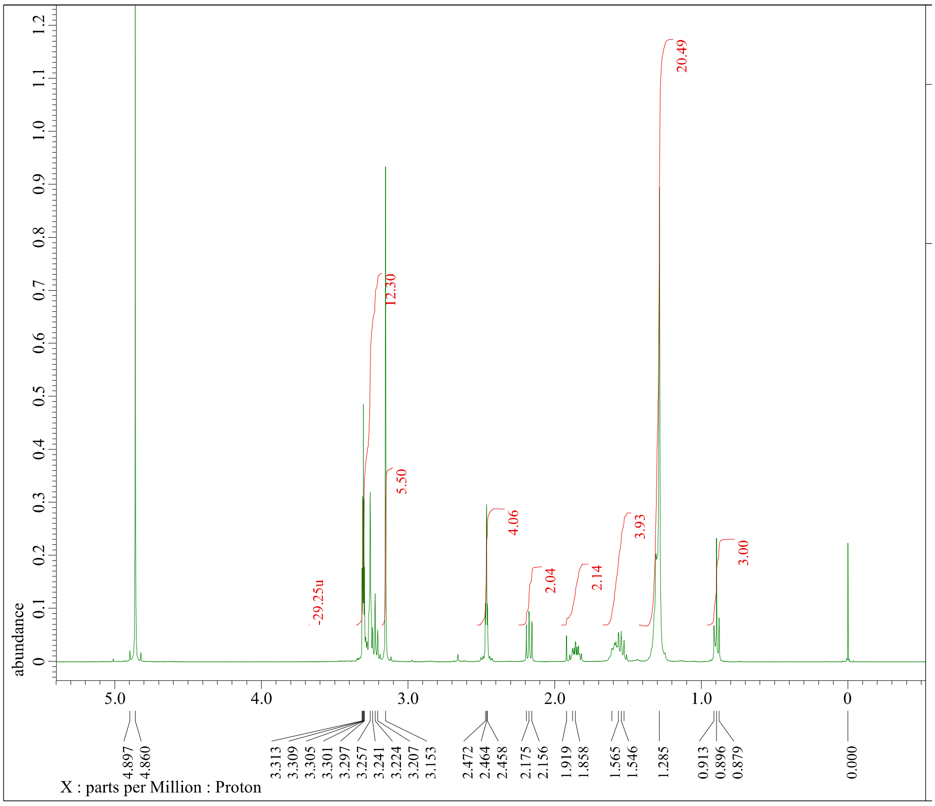

Figure S4. ¹H NMR spectrum of 13-2-2-4.

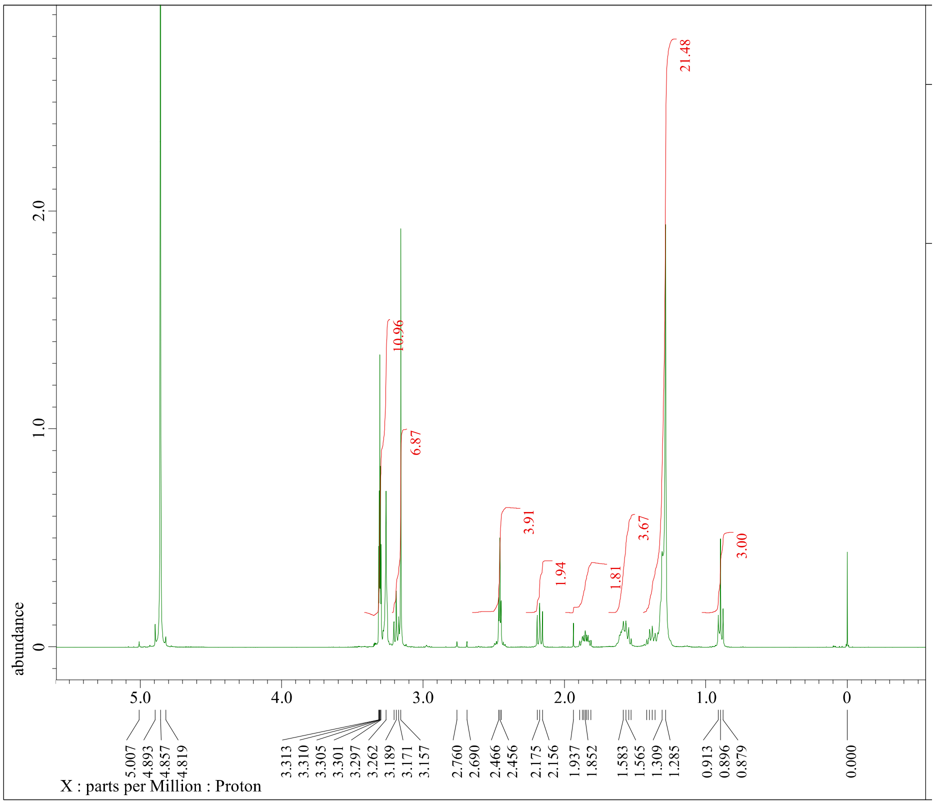

Figure S5. ^{1}H NMR spectrum of 13-2-2-5.

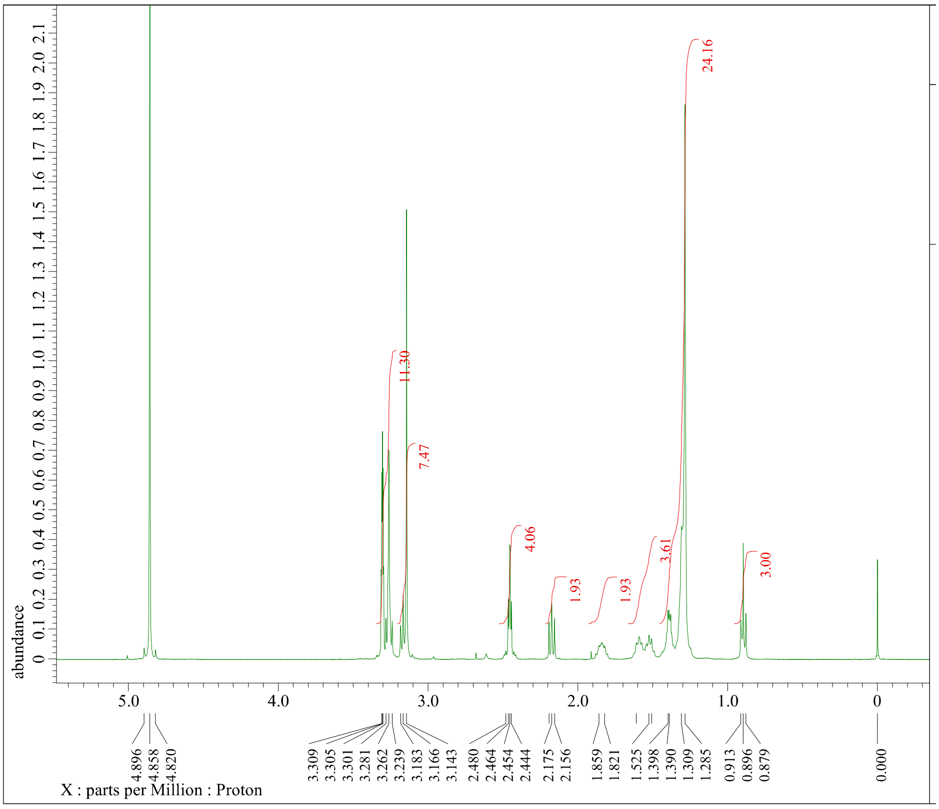

Figure S6. ^{1}H NMR spectrum of 13-2-2-6.

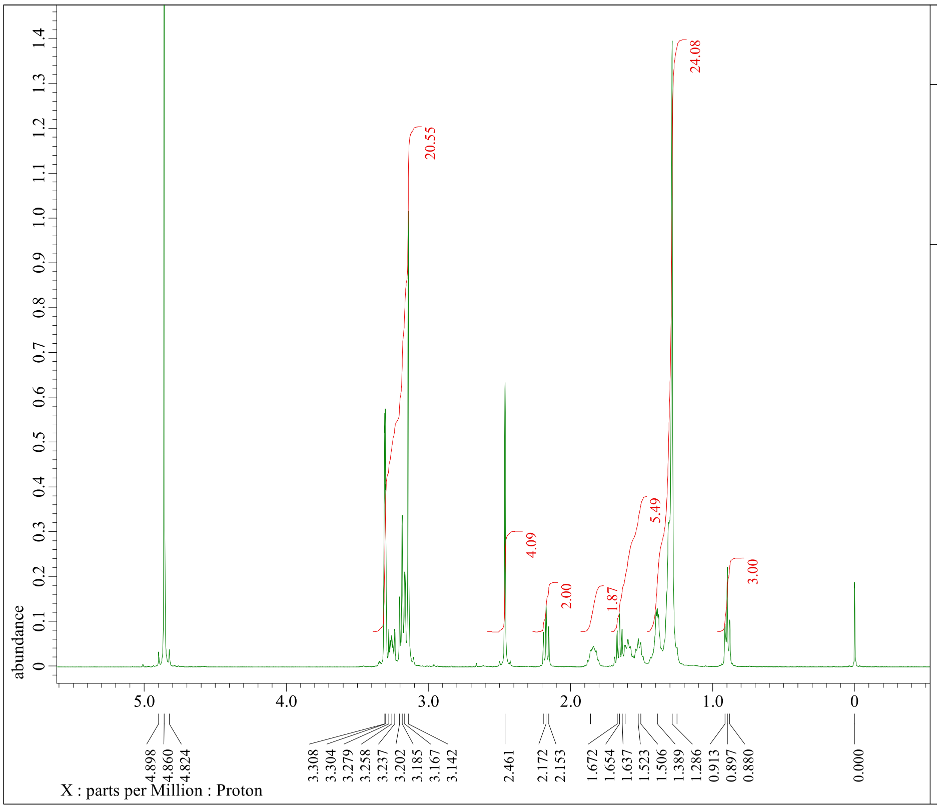

Figure S7. ¹H NMR spectrum of 13-3-2-6.

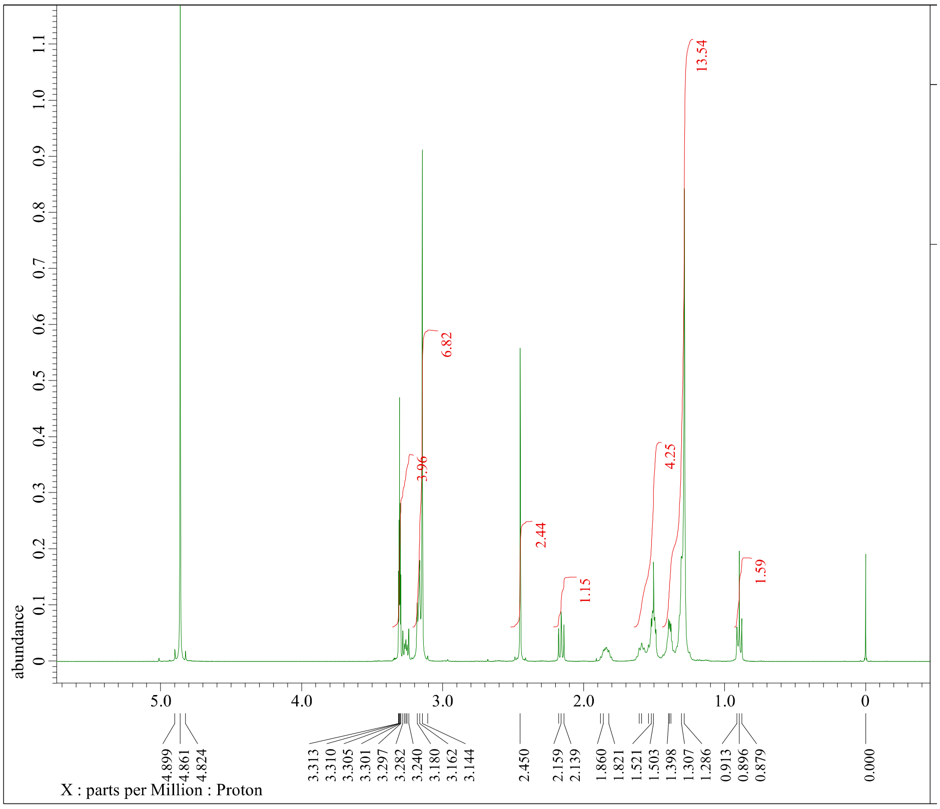

Figure S8. ¹H NMR spectrum of 13-4-2-6.

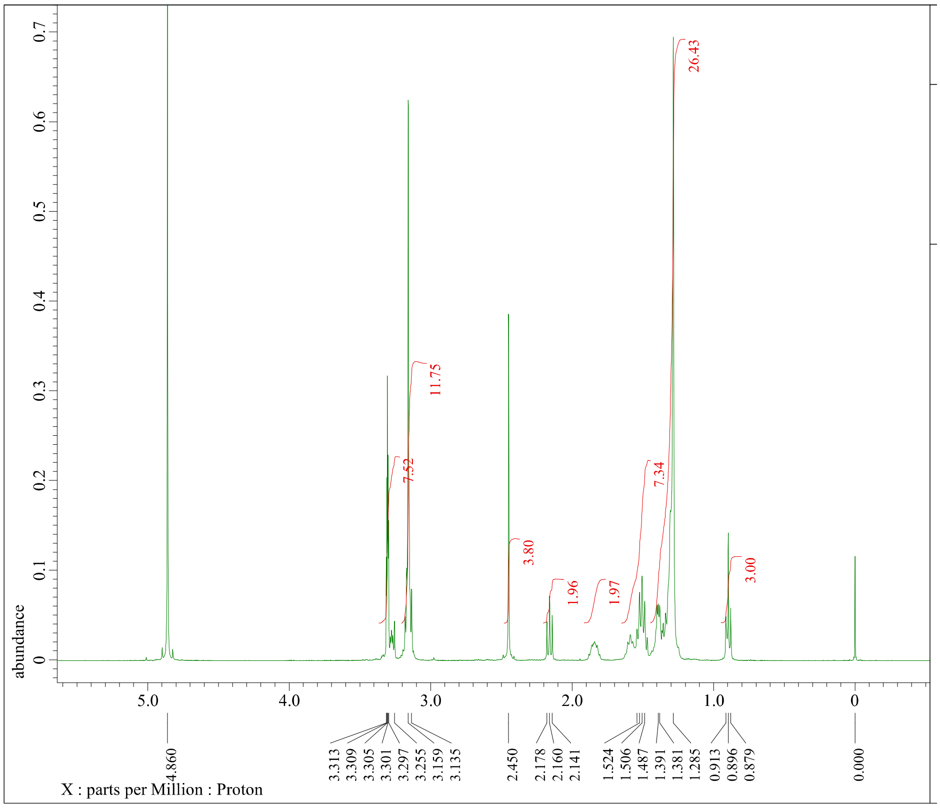

Figure S9. ^{1}H NMR spectrum of 13-5-2-6.

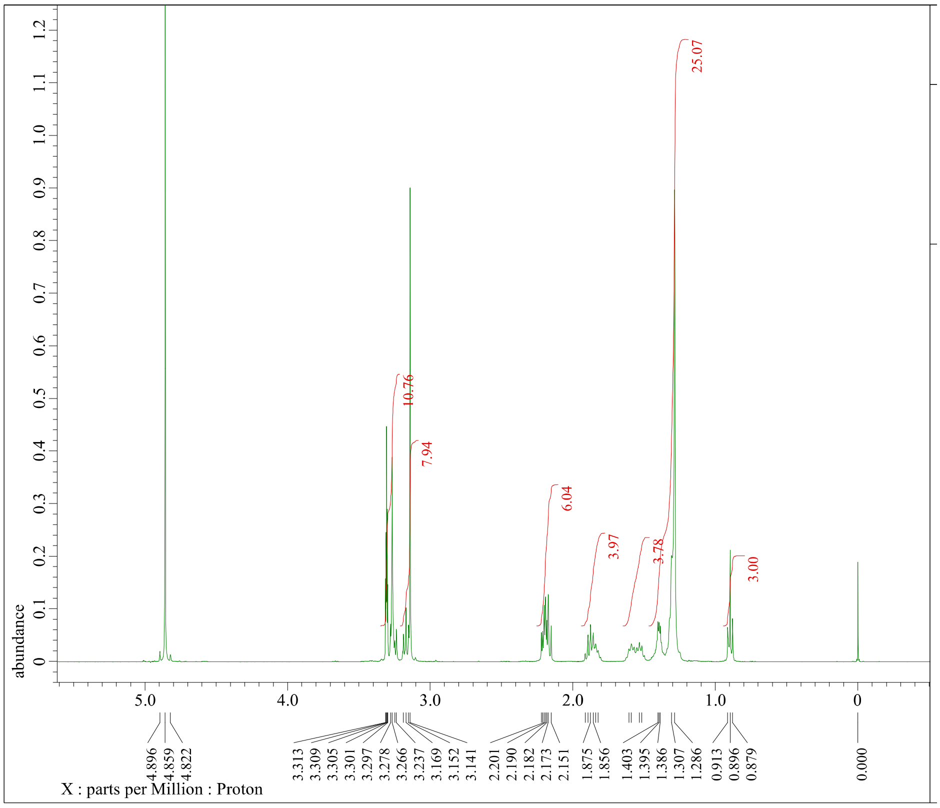

Figure S10. ^{1}H NMR spectrum of 13-2-3-6.
